# Low Prevalence of Anti-DFS70 Antibodies in Children With ANA-Associated Autoimmune Disease

**DOI:** 10.3389/fped.2022.839928

**Published:** 2022-03-22

**Authors:** Mirjam Freudenhammer, Ulrich Salzer, Aileen Heselich, Markus Hufnagel, Ales Janda

**Affiliations:** ^1^Department of Pediatrics and Adolescent Medicine, University Medical Center, Faculty of Medicine, University of Freiburg, Freiburg, Germany; ^2^Center for Chronic Immunodeficiency, University Medical Center, Faculty of Medicine, University of Freiburg, Freiburg, Germany; ^3^IMM-PACT Clinician Scientist Programme, Faculty of Medicine, University of Freiburg, Freiburg, Germany; ^4^Department of Rheumatology and Clinical Immunology, University Medical Center, Faculty of Medicine, University of Freiburg, Freiburg, Germany; ^5^Department of Pediatrics and Adolescent Medicine, University Medical Center Ulm, Ulm, Germany

**Keywords:** DFS70/LEDGF, antinuclear antibodies, autoimmune disease, pediatrics, rheumatology

## Abstract

**Introduction:**

Anti-DFS70 antibodies occur in healthy individuals with various medical conditions. Unlike other anti-nuclear autoantibodies (ANA), they are not associated with systemic autoimmune disease in adult patients. To date, only a few studies have addressed the prevalence and/or clinical relevance of anti-DFS70 autoantibodies in children with and without autoimmune disease.

**Methods:**

Included in this retrospective cross-sectional mono-centric study were 308 pediatric patients with suspected or known autoimmune conditions who had a positive ANA in indirect immune fluorescence (IIF) screening and who were screened for anti-DFS70 antibodies by extractable nuclear antigen antibodies (ENA) immunoblot. Patients were assigned to four different diagnostic categories according to their diagnosis in the corresponding medical record: (a) absence of autoimmune or rheumatic disease (noARD, *n* = 116); (b) suspected autoimmunity without definitive diagnosis (sAI, *n* = 48); (c) other rheumatic disease (ORD) (*n* = 115); and (d) ANA-associated autoimmune disease (AARD, *n* = 29).

**Results:**

The prevalence of anti-DFS70 antibodies in the overall cohort was 33.8%. Among children without ARD (46.6%, 54/116), prevalence was significantly higher than among children with ORD (23.7%, 27/115, *p* = 0.0003) or AARD (17.2%, 5/29, *p* = 0.0054). Among all of the anti-DFS70 positive patients with AARD, other autoantibodies were found in the ENA immunoblot. In contrast, among anti-DFS70 positive patients with ORD (11.5%, 4/27), sAI (33.3%, 6/18) and noARD (16.7%, 9/54), other autoantibodies infrequently were detected (*p* = 0.0005). Patients with uveitis rarely were positive for anti-DFS70 antibodies (7.7%, 1/13). No association was found between anti-DFS70 antibodies and a history of allergic conditions (*p* = 0.51). The concordance between a typical DFS pattern in IIF and the detection of anti-DFS70 antibodies by immunoblot was 59.3%.

**Conclusion:**

As with adults, the higher prevalence of anti-DFS70 among children without autoimmune disease confirms the mutual exclusion for this autoantibody in the pathogenesis of ARD. Among ANA-positive children, monospecific anti-DFS70 antibodies may help to discriminate between AARD and not-AARD-related conditions.

## Introduction

Over 25 years ago, antinuclear autoantibodies (ANAs) presenting a dense fine speckled (DFS) nuclear pattern in indirect immunofluorescence (IIF) assay in HEp-2 cells were first characterized (1). The underlying antigen is known as “lens-epithelium derived growth factor” (LEDGF), due to its detection in a patient with cataracts (2). Later it was designated “DFS70” in reference to its IIF pattern and molecular mass of 70kDa in immunoblots (3). Research suggests that in mammalian cells this ubiquitously present protein acts as a stress activated DNA-binding transcription co-activator. With its multiple DNA/chromatin binding domains it is proposed to be involved in the upregulation of antioxidant stress regulation and inflammatory genes contributing to cellular survival under environmental stress [reviewed in (4, 5)]. Moreover, DFS70/LEDGF plays an important role in different human pathologies. It was recognized to function as an oncoprotein in multiple types of solid cancers (6–10) and hematologic malignancies (11–13) and was shown to be involved in the integration of viral DNA into host chromatin in HIV infection (14, 15). Antibodies against the DFS70 antigen have been identified in a variety of diseases including allergy (3), cancer (6) and inflammatory conditions (1, 16), but they are found also in healthy individuals (17).

In contrast to other ANAs, among adults there is no association between monospecific (i.e., without detection of other ANA-specific antibodies) anti-DFS70 antibodies and ANA-associated rheumatic diseases (AARD), such as systemic lupus erythematosus (SLE), mixed connective tissue disease (MCTD), Sjögren syndrome, systemic sclerosis (SSc) or dermato-/polymyositis (DM/PM) (18–21). Hence, anti-DFS70/LEDGF are increasingly considered as exclusion markers for AARD in adult populations (22–25).

To date, only a few studies have addressed the significance of anti-DFS70 antibodies among children (18, 26–30) (Supplementary Table 1). The reported prevalence of anti-DFS70 antibodies among healthy children ranges between 1.5 and 2% (27, 31). An increased frequency of anti-DFS70 antibodies first was described in children with autoimmune fatigue syndrome (26, 32). Screening a large number of children with AARD and related conditions, Schmeling et al. identified a remarkable increase in frequency of anti-DFS70 antibodies in children with juvenile localized scleroderma (13.8%, 4/29), in patients with juvenile DM (18.2%, 2/11) and in those with uveitis (11.5%, 3/26) (27). Furthermore, they reported that a majority of SLE patients with anti-DFS70 antibodies (68.4%, 13/19) had additional AARD-related antibodies. Increased (and even higher) frequencies of anti-DFS70 antibodies in patients with JDM or uveitis were confirmed by Muro et al. (28); interestingly, however, they did not discover this antibody in children with localized scleroderma. In line with the findings for adult patients with AARD, monospecificity of anti-DFS70 was seen in only 3.4% (1/29) of JDM patients with anti-DFS70 (23).

The aim of our study was to determine the prevalence of anti-DFS70 antibodies in ANA-positive pediatric patients with and without AARD and to analyze their association with other ANA-specific autoantibodies.

## Methods

### Study Population

At the Center for Pediatrics of the University Medical Center Freiburg, Germany, pediatric patients aged ≤ 20 years who had a confirmed positive ANA-IIF screening assay and subsequent ENA-specification with anti-DFS70 between January 2017 and May 2021 were included in this retrospective cross-sectional study. Patients were assigned to one of the following groups according to their diagnosis in the corresponding medical record: (a) no autoimmune or rheumatic disease (noARD): patients with a diagnosis not associated with autoimmunity; (b) suspected autoimmunity (sAI): patients with symptoms suspicious for autoimmunity, but without a definite diagnosis; (c) other rheumatic disease (ORD): patients with an autoimmune disease that does not belong to the group of ANA-associated diseases, i.e., juvenile idiopathic arthritis (JIA), uveitis, ankylosing spondylitis, vasculitis, chronic non-bacterial osteomyelitis (CNBO), type 1 diabetes, localized scleroderma, immune thrombocytopenia, Hashimoto thyroiditis, vitiligo; and d) ANA-associated rheumatic disease (AARD): patients with an autoimmune disease commonly associated with positive ANA-antibodies, i.e., childhood-onset systemic lupus erythematosus (cSLE), mixed connective tissue disease (MCTD), Sjögren's Syndrome, Systemic Sclerosis, undifferentiated connective tissue disease (UCTD), JDM (juvenile dermatomyositis). Of note, we decided to categorize JIA as ORD even though these patients show frequently positivity of ANA antibodies. This arbitrary decision was based on a previously reported classification (27) and an assumption that in contrast to AARD the presence or absence of ANA does not in general change the likelihood that a given patient will have or will develop JIA. Patient-specific clinical data were extracted from the electronic patient record. Conditions such as atopic dermatitis, asthma, or allergies to pollen, food or house dust mites were summarized as “allergic disease”. Other possible comorbidities, duration of the disease and length or type of treatment were not recorded. The study was approved by the Ethics Committee of the University of Freiburg (Nr. 438/18).

### ANA and Autoantibody Assays

ANA-testing of the sera was performed at the diagnostic laboratory in the Department of Rheumatology and Clinical Immunology, University Medical Center Freiburg, Germany. It was conducted via indirect immune fluorescence (ANA-IIF) using Hep2-cells (Inova Diagnostics Inc., San Diego, CA, USA). Titers ≥1:50 were considered positive. The starting dilution of 1:50 was used because the laboratory also offers the same ANA test for evaluation of other ANA related disorders (e.g., autoimmune hepatitis), in which other cut offs apply. ANA slides were viewed at 40 × magnification. The observed immune fluorescence patterns were categorized according to the International Consensus on Autoantibody Patterns ICAP (https://anapatterns.org/index.php) as follows: nuclear [AC-1 (homogenous), AC-2 (dense fine speckled), AC-3 (centromere), AC-4 (fine speckled), AC-5 (large/coarse speckled), AC-6 (multiple discrete nuclear dots), AC-7 (few discrete nuclear dots), AC-8 (nucleolar homogeneous)], cytoplasmic [AC-15 (fibrillar linear), AC-21 (AMA)] or negative/non-reactive (AC-0). The correct assignment to the ICAP classification was crosschecked in direct collaboration with technicians and head of the diagnostic laboratory based on the detailed result reports of ANA IIF on Hep2 cells. Autoantibodies against ENAs (snRNP/Sm, Sm, SS-A/Ro, Ro-52, SS-B/La, Scl-70, PM-Scl, Jo-1, centromere, PCNA, nucleosomes, histones, ribosomal P-protein, AMA-M2) and autoantibodies against DFS70 were detected by immune dot blot (“ANA Profile 3 plus DFS70”, Euroimmun, Lübeck, Germany) at a standard serum dilution of 1:100. Samples scoring “+” or higher (“++”, “+++”) were considered as “positive”. ENA autoantibody testing was performed in each patient with confirmed positive ANA-IIF screening regardless of its pattern. Autoantibodies against dsDNA were identified by ELISA (diagnostik-a GmbH, Ebringen, Germany).

### Statistics

Here we present categorical variables as proportions, while continuous variables are displayed as median and interquartile range. Comparisons among groups were performed using the Mann-Whitney-U-test or Fisher's exact. All analyses were conducted using GraphPad Prism version 8.0.0 for Windows, (GraphPad Software, San Diego, CA USA). A *p-*value of < 0.05 was considered statistically significant.

## Results

### Prevalence of Anti-DFS70 Antibodies in Pediatric Patients With Positive ANA-IIF

We identified 308 pediatric patients with anti-nuclear antibodies in IIF. This included 116 patients without an autoimmune disease, 48 patients with suspected autoimmunity, 115 patients with a non-ANA-associated autoimmune disease and 29 patients with an ANA-associated rheumatic disease. Of these patients, 104/308 (34%) tested positive for anti-DFS70 antibodies by immunoblot. The median age in the two groups was similar with 13 (IQR 7.7) in the anti-DFS70-positive group and 12.2 (8.4) in the anti-DFS70-negative group (*p* = 0.19, Mann-Whitney-U-test). Likewise, no significant difference was found regarding sex ratio with 75/104 (72.1 %) female patients in the anti-DFS70-positive and 153/204 (75%) in the anti-DFS70-negative group (*p* = 0.59, Fisher's Exact test). ANA-titers were reported for 229 patients. Among the anti-DFS70-positive group 23/87 (26.4%) samples had a low ANA-titer (1:100–1:200), 46/87 (52.9%) an intermediate (1:400–1:800) and 18/87 (20.7%) a high titer (≥1:1600). Among the anti-DFS70 negative group the proportions were similar with 41/142 (28.9%) samples revealing a low, 72/142 (50.7%) an intermediate and 29/142 (20.4%) a high ANA-titer.

Among patients without an autoimmune disease, the prevalence of anti-DFS70 as detected by immunoblot was noticeably higher (46.6%, 54/116) than in patients with an ANA- (17.2%, 5/29, *p* = 0.0054) or not-ANA-associated rheumatic disease (23.5%, 27/115, *p* = 0.0003) (Table 1). In the cohorts with an established autoimmune diagnosis, anti-DFS70 antibodies were found in children with a variety of diseases—for example, in patients with JIA (19/85, 22.4%), in those with cSLE (4/17, 23.5%), in those with JDM (1/2, 50%) and in others (Table 1). Only in 1/13 (7.7%) patients with (idiopathic or JIA-associated) uveitis anti-DFS70 antibodies were detected.

**Table 1:** Prevalence of anti-DFS70 antibodies among patients with positive ANA testing in immune fluorescence (IIF).

	*N* ^*^	Anti-DFS70-positive^**^, *n* (%)	***p*-value^**$**^**
**Total patients**	**308**	**104 (33.8)**	
**noARD**	**116**	**54 (46.6)**	
**sAI**	**48**	**18 (37.5)**	**NS**
**ORD**	**115**	**27 (23.7)**	**0.0003**
JIA w/o uveitis	77	18 (23.4)	
JIA w uveitis	8	1 (12.5)	
Idiopathic uveitis	5	0	
Localized scleroderma	2	1	
CNBO	1	1	
ITP	3	2	
DM Type I	2	1	
Hashimoto thyroiditis	2	1	
Vasculitis	1	1	
Vitiligo	1	1	
**AARD**	**29**	**5 (17.2)**	**0.0054**
cSLE	17	4 (23.5)	
MCTD	5	0	
JDM	2	1	
UCTD	2	0	
Sjögren's syndrome	2	0	
Systemic scleroderma	1	0	

* *samples with positive ANA in IIF*.

** *anti-DFS70-positive in immunoblot*.

$ *compared to patients with noARD, Fisher's exact test*.

*AARD, ANA-associated rheumatic disease; ANA, antinucelar antibodies; CNBO, chronic non-bacterial osteomyelitis; cSLE, childhood systemic lupus erythematosus; ITP, immune thrombocytopenia; JIA, juvenile idiopathic uveitis; JDM, juvenile dermatomyositis; MCTD, mixed connective tissue disease; ORD, other rheumatic disease; noARD, no autoimmune or rheumatic disease; sAI, suspected autoimmunity (no diagnosis); UCTD, undifferentiated connective tissue disease; w/o, without; w, with. The bold values represent the superordinate group, the not-bold values the subgroups*.

Information on history of allergic conditions was available for 54.3% of patients. No significant association was found between anti-DFS70 antibodies and allergic disease with a positive history of allergic disease in 23/77 (29.9%) in the anti-DFS70-positive group and 31/89 (34.8%) in the anti-DFS70-negative group (*p* = 0.51, Fisher's Exact test).

### Association of ENAs or Anti-dsDNA Antibodies Among Patients With Anti-DFS70 Antibodies

In the anti-DFS70-positive group, only 4.8% (5/104) of patients had an AARD (4 with SLE, 1 with JDM), and all five patients had at least one additional disease-specific antibody, (anti-dsDNA, anti-snRNP, anti-SS-A, anti-Ro-52, anti-nucleosome, anti-histone, anti-ribosomal P-Protein and/or anti-AMA-M2) (Table 2). By contrast, disease-associated antibodies were uncommon in anti-DFS70 positive children without AARD, (19/99, *p* = 0.0005).

**Table 2:** Associated antibodies in anti-DFS70-positive patients.

	**No ARD**	**sAI**	**ORD**	**AARD**	*p*-value^*^
	***n* = 54**	***n* = 18**	***n* = 27**	***n* = 5**	
**any other antibody** ***n*** **(%)**	**9 (16.7)**	**6 (33.3)**	**4 (14.8)**	**5 (100)**	**0.0005**
anti-dsDNA, *n* (%)	0	0	1 (3.7)	3 (60)	
anti-snRNP/Sm, *n* (%)	0	0	0	1 (20)	
Anti-Sm, *n* (%)	0	0	0	0	
anti-SS-A/Ro, *n* (%)	1 (1.9)	0	0	1 (20)	
anti-Ro-52, *n* (%)	0	0	0	0	
anti-SS-B/La, *n* (%)	0	0	0	0	
anti-Scl-70, *n* (%)	0	0	0	0	
anti-PM-Scl, *n* (%)	3 (5.6)	0	0	0	
anti-Jo-1, *n* (%)	0	0	0	0	
anti-Centromere, *n* (%)	2 (3.7)	2 (11.1)	1 (3.7)	0	
anti-PCNA, *n* (%)	1 (1.9)	2 (11.1)	1 (3.7)	0	
anti-Nucleosome, *n* (%)	2 (3.7)	1 (5.6)	1 (3.7)	2 (40)	
anti-Histone, *n* (%)	1 (1.9)	1 (5.6)	0	1 (40)	
anti-ribosomal P-Protein, *n* (%)	0	0	0	1 (40)	
anti-AMA-M2, *n* (%)	1 (1.9)	0	0	0	

* *AARD tested against “No AARD” groups, Fisher's exact-test*.

*AARD, ANA-associated rheumatic disease; ORD, other rheumatic disease; noARD, no autoimmune or rheumatic disease; sAI, suspected autoimmunity (no diagnosis). The bold values represent the superordinate group, the not-bold values the subgroups*.

### Concordance of DFS Pattern in IIF-ANA With Anti-DFS70 Antibodies in Immunoblot

In examining the agreement of a DFS pattern (AC-2) in IIF with anti-DFS70 antibodies in immunoblot, we detected anti-DFS70 antibodies in 59.3% (54/91) of samples with a dense fine speckled pattern in indirect immunofluorescence (Table 3). The most common pattern among anti-DFS70 positive samples was the nuclear DFS-pattern (54/104, 51.9%), while a nuclear homogenous pattern was predominant among the anti-DFS negative samples (92/204, 45.15%) (Table 3).

**Table 3:** Agreement between IIF pattern (Hep2-cells) and anti-DFS70 antibodies in immunoblot.

**IIF pattern**	**Anti-DFS70-positive *n* = 104**	**Anti-DFS70-negative *n* = 204**
AC-0 (negative)	0	5 (2.5)
AC-1 (homogenous), *n* (%)	39 (37.5)	92 (45.1)
AC-2 (dense fine speckled), *n* (%)	54 (51.9)	37 (18.1)
AC-3 (centromere), *n* (%)	1 (1.0)	6 (2.9)
AC-4 (fine speckled), *n* (%)	10 (9.6)	33 (16.2)
AC-5 (large/coarse speckled), *n* (%)	0	17 (8.3)
AC-7 (few discrete nuclear dots), *n* (%)	4 (3.8)	1 (0.5)
AC-8 (nucleolar homogeneous), *n* (%)	1 (1.0)	10 (4.9)
AC-15 (fibrillar linear), *n* (%)	0	7 (3.4)
AC-21 (AMA), *n* (%)	0	6 (2.9)

*AMA, anti-mitochondrial antibodies. The bold values represent the superordinate group, the not-bold values the subgroups*.

## Discussion

Previous studies of adult patients have suggested that healthy individuals have a higher frequency of anti-DFS70 antibodies than patients with systemic autoimmune diseases (17, 19). To our knowledge, ours is the first study to be able to confirm these findings for pediatric patients. Specifically, we show that ANA-positive pediatric patients with an ANA-associated rheumatic disease have a lower prevalence of anti-DFS70 antibodies than patients without an autoimmune or rheumatic disease (17.2 and 46.6%, respectively). However, especially regarding pediatric patients, comparability of anti-DFS70 frequencies with other studies may be limited. In our setting, ANA-positive patients were preselected prior to testing for anti-DFS70 antibodies. Furthermore, it should be noted that our “noARD” cohort is not equivalent to a healthy cohort, since those patients may have suffered from conditions other than autoimmune or rheumatic diseases. An Italian study of 261 healthy school children reported anti-DFS70 antibodies in 1.5% of samples. When calculated for ANA-positive samples, a frequency of 12.5% anti-DFS70 antibody positivity among healthy ANA-positive children may be determined (31). Schmeling et al. (27) found anti-DFS70 antibodies in only 2.1 % of healthy children. When stratifying ANA-positive samples of their “query connective tissue disease” cohort (i.e., children with suspicion of CTD), they observed an anti-DFS70 frequency of 16.4%, which is comparable to the AARD cohort from our study (16.7%). A recent Chinese study reported a DFS pattern in 10.6% of ANA-positive sera from pediatric patients undergoing routine ANA-testing (30). Among adult patients, the reported prevalence of anti-DFS70 antibodies in ANA-positive cohorts ranges broadly from 4.6 to 54% (17, 22, 33–36). Presumably this is due to differences in methodology and patient selection criteria.

As in the case of adult patients, the clinical relevance of anti-DFS70 antibodies in pediatric patients is unknown. In the above-mentioned study with healthy Italian schoolchildren, no development of AARD among anti-DFS70 positive pediatric patients was observed during the three-year follow-up (29). Anti-DFS70 antibodies were described in pediatric patients with a range of autoimmune diseases, including localized scleroderma, juvenile dermatomyositis and uveitis (27, 28). Our study detected anti-DFS70 antibodies in a variety of autoimmune diseases, including JIA (22.4%) and cSLE (23.5%), but the frequency was lower than in ANA-positive patients without autoimmune or rheumatic disease (46.6%). This argues against a disease-specific role for this antibody. In fact, given the high prevalence of anti-DFS70 antibodies in ANA-positive healthy individuals, some authors even proposed a protective role for this antibody (18, 37, 38). This idea is supported by studies in adult SLE patients that reported lower disease activity in anti-DFS70 positive SLE patients (19, 21) and by a study in patients with dermatomyositis complicated by interstitial lung disease (including one patient with juvenile DM) where anti-DFS70 antibody levels increased upon disease remission (18). In addition, in animal experiments with lupus-prone mice improved survival rates were demonstrated after weekly infusions of anti-DFS70 antibodies, implicating anti-DFS70 antibodies as a possible therapeutic approach (39). Recently, another study indicated an association of higher vitamin D levels with the presence of anti-DFS70 antibodies (40), taking into consideration the probable connection of vitamin D deficiency and autoimmunity (41). Yet another study reported the induction of anti-DFS70 antibodies under anti-TNFα treatment (42). However, further longitudinal studies are needed to address the question about a possible protective nature of anti-DFS70 antibodies.

In contrast to other reports (27, 28), in our study cohort, we were unable to detect an increased prevalence in anti-DFS70 antibodies among pediatric patients with (idiopathic or JIA-associated) uveitis (7,7%) as compared to patients without autoimmune disease (46,6%) or as compared to those with JIA but without uveitis (23,4%). This may be due to our study's preselection on ANA-positive patients, as well as to the generally lower prevalence of antinuclear antibodies in healthy children (29, 43) and/or in JIA patients without uveitis, as compared to JIA patients with uveitis (44, 45) or CTD (46–48).

Although the data are conflicting, previous studies have suggested an association between anti-DFS70 antibodies and atopic conditions (3, 19). Our results do not support an association between the occurrence of anti-DFS70 antibodies and a history of allergic conditions.

In line with findings for adult patients (19, 35, 49), the diagnosis of AARD among patients with a positive anti-DFS70 antibody test was low (4.8%, 5/104). Furthermore, in all five AARD patients with a positive anti-DFS70 result, associated disease-specific antibodies were detected. By contrast, in our cohort, a single anti-DFS70 positivity (“monospecificity”) was found in 83% of pediatric patients without autoimmune disease. This indicates that, as with the adult population, anti-DFS70 antibodies in children with AARD are rare (19, 23). This is in accordance with Muro et al., who report that only 1 of 8 JDM patients with anti-DFS70 antibodies had no other autoantibodies (28).

Due to the low prevalence of monospecific anti-DFS70 in adult patients with AARD, several authors have suggested including tests for anti-DFS70 antibodies in the diagnostic approach for suspected AARD (24, 25, 35, 49–55). A recent systematic review and meta-analysis showed a high specificity for anti-DFS70 antibodies to exclude AARD among patients with a positive ANA-IIF (56). Our results indicate that it might be of use to apply this in pediatric settings as well. However, our study's patient numbers were too low to provide reliable predictive values on this point. For this purpose, larger studies will be needed.

In our study, the agreement between IIF and immunoblot for anti-DFS70 antibodies was 59%. This is in accordance with the rates reported by other surveys (30–>90%) (19, 27, 30, 33, 49, 57–59). One-third of patients with a DFS pattern in IIF did not have anti-DFS antibodies in the consecutive immunoblot. In addition, a DFS pattern in IIF was detected in only half of patients with detectable anti-DFS70 antibodies. The discrepancies between IIF and DFS-specific immunoassays might partly be the result of difficulties recognizing the DFS pattern by conventional Hep2-IIF methods. An international internet-based survey demonstrated an accuracy of only 50% in correct identification of the unmixed DFS pattern, significantly lower than other classical IIF patterns (60). Furthermore, autoantibodies other than anti-DFS70 might produce a DFS-like pattern in samples that are negative in anti-DFS specific tests. Possible antigens for these autoantibodies are cellular proteins interacting with DFS70/LEDGF in a macromolecular transcription complex like MeCP2, MLL1 or Menin (38, 61, 62). Our findings support the need for additional tests (e.g., immunoblot or ELISA) in order to confirm anti-DFS70 antibody status among pediatric patients, as has been suggested by reports showing low accuracy in the identification of anti-DFS70 antibodies by ANA-IIF-only among adults (58, 60, 63).

The study's retrospective nature imposes potential limitations on the precision with which disease/non-disease cohort assignments can be made and, on the inability, to validate autoantibody assay results independently. Additionally, stratification for disease duration, co-morbidities, and treatments was not possible and thus introduction of confounding bias cannot be excluded. Therefore, the results of the study are preliminary and the findings require confirmation in a future prospective study with an inception cohort.

## Summary and Conclusion

Our study indicates that anti-DFS70 antibodies in children play a similar role as among adults. We found a lower prevalence of anti-DFS70 antibodies among ANA-positive children with a confirmed diagnosis of AARD or ORD, as compared to children without autoimmune disease. This largely excludes a specific role for this antibody in the pathogenesis of pediatric AARD. When occurring in children with AARD, anti-DFS70 were accompanied by additional disease-specific antibodies. By comparison, among children without AARD, monospecific anti-DFS70 were common. Additional studies are needed in order to investigate if determination of anti-DFS70 antibodies in a child with a positive ANA-IIF and without associated disease-specific autoantibodies may help us to more effectively discriminate between cases where AARD is present and those where children are either healthy or else have a non-ANA associated condition.

## Data Availability Statement

The raw data supporting the conclusions of this article will be made available by the authors, without undue reservation.

## Ethics Statement

The studies involving human participants were reviewed and approved by the Ethics Committee of the University of Freiburg (Nr. 438/18). Written informed consent from the participants' legal guardian/next of kin was not required to participate in this study in accordance with the national legislation and the institutional requirements.

## Author Contributions

MF: data collection and analysis and manuscript writing. US: laboratory data collection and analysis and review and editing of the manuscript. AH: data collection and analysis. MH: study concept, supervision of work, and review and editing of the manuscript. AJ: study concept, data collection, supervision of work, and review and editing of the manuscript. All authors contributed to the article and approved the submitted version.

## Funding

MF is supported by the German Research Council with an IMM-PACT Clinician Scientist fellowship (413517907).

## Conflict of Interest

The authors declare that the research was conducted in the absence of any commercial or financial relationships that could be construed as a potential conflict of interest.

## Publisher's Note

All claims expressed in this article are solely those of the authors and do not necessarily represent those of their affiliated organizations, or those of the publisher, the editors and the reviewers. Any product that may be evaluated in this article, or claim that may be made by its manufacturer, is not guaranteed or endorsed by the publisher.
