# Supplementary material for: Low Prevalence of Anti-DFS70 Antibodies in Children With ANA-Associated Autoimmune Disease
Source: Front Pediatr. 2022 Mar 22;10:839928. doi: 10.3389/fped.2022.839928 (PMC8980602; doi:10.3389/fped.2022.839928)
Supplement: Supplementary file 1 [file Table_1.pdf]

Supplementary Material

Supplementary Table 1 **Published studies reporting on anti-DFS70 antibodies in children.**

Citations	Study group	Key results	Comments
Itoh *et al.* [26]	140 Japanese children with chronic non-specific complaints, 82 healthy control children	ANA-positivity in 52.9% of symptomatic and in 6.1% of healthy control children. 41.3% of ANA-positive sera from children with chronic non-specific complaints had "anti-Sa" antibodies	"anti-Sa" was later recognized to be identical with anti-DFS70 [19]
Schmeling *et al.* [27]	743 children with AARD and related conditions from 9 pediatric medical centers in Germany, Canada and USA, reference cohorts of 145 healthy children and 200 children referred for ANA-testing as part of investigations for AARD	Anti-DFS70 in 2.1% (3/145) of healthy children and in 4.5% (9/200) of children referred for ANA-testing, in 13.8 % (4/29) of children with juvenile localized scleroderma, in 18.2% (2/11) with JDM, in 5.7% (19/331) with cSLE, in 4.5% (1/22) with Systemic sclerosis, in 2.5% (5/202) with JIA and in 11.5% (3/26) of children with uveitis/JIA-associated uveitis.<br>In 6/19 (31.6%) anti-DFS70 positive cSLE patients no other autoantibodies were detected.	
Muro *et al.* [18]	116 Japanese patients with DM, 13 of these with JDM	Anti-DFS70 antibodies were found in 15% (2/13) of patients with JDM	
Muro *et al.* [28]	29 Japanese children with JDM, 14 with localized scleroderma, 10 with JIA	Anti-DFS70 in 27.6% (8/29) patients with JDM, only 1 (3.4%) with monospecific anti-DFS70.	
Sperotto *et al.* [29]	261 healthy Italian school children	ANA-positivity in 32/261 (12.3%) children, 4/261 (1.5%) were anti-DFS70-positive, no ENA- or anti-dsDNA-positivity. In a three year-follow up 92.9% of ANA-positive subjects remained ANA-positive, none of the subjects developed SARD. None of anti-DFS70-negative children turned anti-DFS70 positive, 1/3 anti-DFS70-positive children became anti-DFS70 negative (one was lost to follow up).	
Zheng *et al.* [30]	20293 adult and 756 pediatric Chinese patients undergoing routine HEp-2 IFA screening, 4234 healthy adult individuals	348/756 (46.9%) pediatric patients were HEp2-IFA positive, of these 37/348 (10.6%) had a DFS pattern, compared to 3.3% of HEp2-IFA positive sera from adult patients.<br>20/40 (50%) pediatric patients with anti-DFS70 antibodies in ELISA and LIA were diagnosed with SARD (9 JIA, 8 UCTD, 3 SLE).	

ANA: antinucelar antibodies; *AARD:* ANA-associated rheumatic disease; *cSLE*: childhood systemic lupus erythematosus; *DFS:* dense fine speckled; *ENA*: extractable nuclear antigen; *JIA:* juvenile idiopathic uveitis*; JDM*: juvenile dermatomyositis; SARD: systemic autoimmune rheumatic disease; *UCTD*: undifferentiated connective tissue disease.
